# Distinct sets of olfactory receptors highly expressed in different human tissues evaluated by meta-transcriptome analysis: Association of OR10A6 in skin with keratinization

**DOI:** 10.3389/fcell.2023.1102585

**Published:** 2023-01-26

**Authors:** Shinobu Nakanishi, Taiki Tsutsui, Nao Itai, Mitsuhiro Denda

**Affiliations:** ^1^ Shiseido Global Innovation Center, Yokohama, Japan; ^2^ Institute for Advanced Study of Mathematical Sciences, Meiji University, Tokyo, Japan

**Keywords:** weight gene correlation network analysis (WGCNA), c3net analysis, olfactory receptor (OR), keratinocytes, skin

## Abstract

Olfactory receptors (ORs) are expressed in many tissues and have multiple functions. However, most studies have focused on individual ORs. Here, we aimed to conduct a comprehensive meta-transcriptome analysis of OR gene expression in human tissues by using open-source tools to search a large, publicly available genotype-tissue expression (GTEx) data set. Analysis of RNA-seq data from GTEx revealed that OR expression patterns were tissue-dependent, and we identified distinct sets of ORs that were highly expressed in 12 tissues, involving 97 ORs in total. Among them, *OR5P2*, *OR5P3* and *OR10A6* were associated with skin. We further examined the roles of these ORs in skin by performing weighted gene correlation network analysis (WGCNA) and c3net analysis. WGCNA suggested that the three ORs are involved in epidermal differentiation and water-impermeable barrier homeostasis, and *OR10A6* showed the largest gene sub-network in the c3net network. Immunocytochemical examination of human skin keratinocytes revealed a sparse expression pattern of OR10A6, suggesting that it is not uniformly distributed among all keratinocytes. An OR10A6 agonist, 3-phenylpropyl propionate (3PPP), transiently increased intracellular Ca^2+^ concentration and increased cornified envelope (CE) production in cultured keratinocytes. Knock-down of OR10A6 diminished the effect of 3PPP. Overall, integration of meta-transcriptome analysis and functional analysis uncovered distinct expression patterns of ORs in various human tissues, providing basic data for future studies of the biological functions of highly expressed ORs in individual tissues. Our results further suggest that expression of OR10A6 in skin is related to epidermal differentiation, and OR10A6 may be a potential target for modulation of keratinization.

## Introduction

Recent developments in open-source analysis tools and public data repositories have provided new opportunities for rapid and inexpensive analysis of large-scale human data sets. Here, we take advantage of the genotype-tissue expression (GTEx) public data set (Consortium, 2013) to investigate the tissue expression patterns of protein-coding olfactory receptor (OR) genes. OR genes account for approximately 50% of G protein-coupled receptor genes, which constitute the largest group in human membrane receptors (Naressi et al., 2022). ORs are known to be distributed at many tissues, such as lung, heart, kidney, brain, prostate, airway, and testis (Kang and Koo, 2012; Massberg and Hatt, 2018; Oh, 2018; Lee et al., 2019; Raka et al., 2021). ORs have a wide range of functions, and altered OR function is associated with various diseases such as cancer, myelogenous leukemia, and retinitis (Raka et al., 2021). Evolutionary analysis also supports the idea that ORs expressed in each tissue have distinctive functions in non-olfactory tissues (De la Cruz et al., 2009), and they are considered promising candidates as therapeutic targets. Recent studies suggest that ORs might play a variety of roles in the *epidermis*. For example, activation of OR2AT4 by sandalore accelerates wound healing in human *epidermis* (Busse et al., 2014), while activation of OR2A4/7 and OR51B5 influences cell proliferation and migration, respectively (Tsai et al., 2017). OR51E2 is expressed in melanocytes, and its activation enhances melanogenesis (Gelis et al., 2017). Involvement of OR10G7 in atopic dermatitis is also reported (Tham et al., 2019). However, studies in non-olfactory tissues have mainly focused on individual ORs.

In this work, we aimed to comprehensively define the distinct expression patterns of OR genes in different human tissues by means of GTEx meta-transcriptome analysis. Based on our research interests, we then examined the role of ORs whose expression is associated with skin by employing weight gene correlation network analysis (WGCNA) and c3net analysis. Our network analysis indicated that the three ORs highly expressed in skin compared to other tissues all belong to the same gene co-expression module, which contains genes associated with epidermal differentiation and water-impermeable barrier homeostasis. Functional and immunohistochemical studies further suggested that one of them, OR10A6, is related to keratinization.

## Materials and methods

### Meta-transcriptome analysis

We employed GTEx project version 8 (https://gtexportal.org/home/datasets). In this study, we used already-processed transcripts per million (TPM) per gene data for further data processing. The metadata was also obtained from the GTEx project. For the present purpose, we removed several tissues for which less than 100 samples were available. In total, 17,244 samples from 28 different tissues were used for the following analysis.

In the case of samples annotated as skin samples containing both skin tissue and cultured fibroblasts, we removed the fibroblast data from the skin tissue data and re-categorized it as “cultured fibroblast”. In this analysis, we employed OR gene definitions from KEGG pathway data (hsa04740) and Reactome (R-HSA-381753). These pathways contain non-OR genes, and so we used genes for which the gene symbol starts with “OR” and does not have “*p*” at the end (to exclude pseudogenes). This yielded 404 possible protein-coding OR genes.

Before analyzing the distinct patterns of tissue expression of these OR genes, we used uniform manifold approximation and projection (UMAP) to visualize all samples (Konopka, 2022) to see if samples were clustered by tissue. Next, to find distinct sets of OR genes highly expressed in different tissues, we combined the Wilcoxon rank sum test, fold expression and linear discriminant analysis (LDA). Statistics were calculated for each OR in a tissue-by-tissue manner. In this study, we define tissue-associated ORs using the following criteria: *p*-value <10^-10, fold change≥25, and area under curve for the LDA receiver operating characteristic (ROC) curve≥0.85.

### Weighted gene correlation network analysis (WGCNA)

To examine possible functional interactions between OR genes and biological processes in skin tissue, we employed WGCNA according to the manual (Langfelder and Horvath, 2008). In line with our research interests, we focused especially on skin tissue. We obtained all skin samples without fibroblasts, amounting to 1305 skin tissues, including 604 samples of sun-protected and 701 samples of sun-exposed skin. Genes for which expression was not detected at least 650 samples were removed. A value of 0.0001 was added to all gene’s TPM before log10 transformation. Soft-thresholding power for WGCNA analysis was determined using pickSoftThreshold function in WGCNA package using “signed” network setting. Estimated power was used for adjacency calculation at “signed” network setting and network topological overlap matrix (TOM) similarity was calculated using the “unsigned” setting. Gene modules were detected by hierarchical clustering and closely related modules were merged according to the user’s manual. For simple interpretation of the TOM network, we tried network visualization after the removal of TOM less than 0.025. The network was visualized using Cytoscape.

To visualize fold gene expression levels in skin tissue, we first calculated average gene expression per tissue and then calculated the average for all tissues. Log2-transformed values were used for visualization.

### C3net analysis

For further insight into the OR gene-containing network module, we employed c3net analysis (Altay and Emmert-Streib, 2010). C3net utilize maximum mutual information (MI) for gene networking. Here we used the c3net package in R-language for detailed segmentation of the WGCNA gene network module.

### Gene ontology (GO) term enrichment analysis

For functional insight into WGCNA network module, functional enrichment was performed in GO terms: Biological process to each gene module using clusterProfiler packages (Yu et al., 2012). A corrected *p-value* (*q-value*)≤0.05 was chosen as the threshold for significantly enriched GO terms. In addition, all GO categories have a gene count of 10 or greater.

### Cells and cell culture

Normal human epithelial keratinocytes were purchased from Kurabo (Osaka, Japan) and cultured in EPILIFE-KG2 (Kurabo, Osaka, Japan). Keratinocytes were seeded onto collagen-coated glass coverslips (Matsunami, Osaka, Japan) and used within 4 days. Keratinocytes were first cultured to 100% confluency in 0.06 mM Ca^2+^ medium with or without siRNA for 24 h and then used for experiments.

### Quantitative real-time PCR (RT-PCR)

Total RNA from human keratinocytes was isolated using a RNeasy mini kit (QIAGEN, Hilden, Germany), and complementary DNA (cDNA) synthesis was performed from 1 μg of total RNA using SuperScript VILO Master Mix (Invitrogen, Carlsbad, United States of America). The PCR reactions were performed using LightCycler 480 Probes Master (Roche, Basal, Switzerland), cDNA and specific primer pairs: *GAPDH*: forward, gaa​ggt​gaa​ggt​cgg​agt​c and reverse, gaa​gat​tgg​tga​tgg​gat​ttc; *OR5P2*: forward, acc​ttc​att​tat​gtg​atg​c and reverse, aaa​ata​aca​agc​atc​atg​ag; *OR5P3*: forward, cag​tca​ctc​tgt​tct​atg​g and reverse, taa​gct​ctc​tct​tca​gag​c; *OR10A6*: forward, tat​tta​caa​ccc​aaa​tct​g and reverse, tca​gat​tgt​gtg​taa​aac​c, on an LightCycler 480 System II (Roche, Basel, Switzerland). Results were normalized with respect to the GAPDH gene.

### siRNA transfection

The cells were grown to 80% confluency, and transfected with 20 nM scramble control or OR10A6 siRNA (GE Dharmacon, Lafayette, United States of America) using the transfection reagent RNA iMAX (Thermo Fisher Scientific, Waltham, United States of America) in OptiMem (Thermo Fisher Scientific) as described in the manual. Scramble control: ugg​uuu​aca​ugu​cga​cua​a, ugg​uuu​aca​ugu​ugu​gug​a, ugg​uuu​aca​ugu​uuu​cug​a and ugg​uuu​aca​ugu​uuu​ccu​a. OR10A6 siRNA: guu​caa​aca​uca​ugg​gua​u, ccg​gaa​acc​aag​aaa​gug​a, gaa​auu​aug​gcg​aag​gcg​a and ugg​cuu​ucc​ugg​uua​uuu​a.

### Ratiometric fluorescence measurement of intracellular calcium

Changes of intracellular calcium concentration in single cells were measured with Fura-2 AM according to the manufacturer’s instructions (Molecular Probes Inc., Eugene, United States of America). Briefly, cells were loaded with 5 μM Fura-2 AM at 37°C for 45 min. After loading, the cells were rinsed with balanced salt solution containing (in mM): NaCl 150, KCl 5, CaCl_2_ 1.8, MgCl_2_ 1.2, HEPES 25, and D-glucose 10 (pH 7.4), abbreviated as BSS(+), and incubated for a further 10 min at room temperature to allow de-esterification of the loaded dye.

The coverslip was mounted on an inverted epifluorescence microscope (ECLIPSE Ti, Nikon, Tokyo, Japan), equipped with a 75 W xenon lamp and band-pass filters of 340 and 380 nm. Imaging was done with a high-sensitivity CCD camera (ORCA-R2, Hamamatsu Photonics, Hamamatsu, Japan) under the control of a Ca^2+^ analyzing system (AQUACOSMOS/RATIO, Hamamatsu Photonics). The intracellular calcium concentration was measured every second with or without addition of 3-phenylpropyl propionate (3PPP) (50 μM, 500 μM or 1 mM), SQ-22536 (100 μM) or L-*cis*-diltiazem (100 μM). Addition of 3PPP, 3PPP + SQ-22536 mixture and 3PPP + L-*cis*-diltiazem mixture were performed by using reflux system (VC-6 SIX CHANNEL VALVE CONTROLLER, Hamamatsu Photonics, Hamamatsu, Japan).

### cAMP measurement

Keratinocytes were cultured to confluence in 96-well culture plates and then treated with 3PPP (1 mM) or ethanol (control) with or without addition of SQ-22536 (100 μM) or L-*cis*-diltiazem (100 μM) dissolved in medium for 15 min at 37°C. cAMP level in the cells was analyzed by using cAMP-Glo™ Max Assay (Promega, Wisconsin, United States of America) according to the manufacturer’s instructions. The luminescence was detected with a microplate reader (SYNERGY H1, BioTek, Vermont, United States of America).

### Cornified envelope (CE) count assay

CE count assay was conducted as described previously (Sun and Green, 1976; Denda et al., 2012). Keratinocytes were cultured to confluence with or without OR10A6 siRNA in 24-well culture plates and then differentiated in 1.8 mM Ca^2+^ medium for 3 days 3PPP (1 mM) was diluted with ethanol and added to 1.8 mM Ca^2+^ medium. The same amount of ethanol was added to 1.8 mM Ca^2+^ medium as the control. All wells were sealed with polyolefin micro sealing tape (3 M Japan Limited, Tokyo, Japan) to prevent evaporation. After 3 days, cells were washed with HBS buffer and treated with trypsin. To halt trypsinization, Trypsin Neut Solution (Kurabo Industries Ltd., Osaka, Japan) was added, and cells were stained with trypan blue solution (Nacalai tesque Inc., Kyoto, Japan). After viable cell counting, samples were centrifuged (15,000 rpm, 5 min) and the supernatant was discarded. To lyse cells, lysis solution (20 mM Tris-HCl (pH 7.5), 1% β-mercaptoethanol, 1% SDS) was added to the samples and the mixtures were incubated at 95°C for 15 min. Residual CE was counted under a microscope. The CE production rate was calculated as a percentage of that of viable cells.

### Human phosphokinase array analysis

Keratinocytes were cultured to confluence in 6-well culture plates and then treated with 3PPP (1 mM) or ethanol (control) dissolved in 1.8 mM Ca^2+^ medium for 15 min at 37°C. After that, cell lysates were prepared according to the manufacturer’s instruction, and analyzed using a Human Phospho-Kinase Array kit (R&D Systems, Minnesota, United States of America). Semi-quantitation was performed by densitometry (Fusion FX7, Vilber Bio Imaging, Collégien, France) (*n* = 4). The signal densities were corrected on the basis of positive reference signals.

### Histology

Cultured cells were fixed with 4% paraformaldehyde in PBS for 15 min and human skin samples were fixed with acetone at −20°C for 30 min, embedded in paraffin, and sectioned at 3 µm. Immunostaining was performed with antibody to OR10A6 (DF5047, Affinity Biosciences, Cincinnati, United States of America), KRT14 (20 R-CP002, Fitzgerald, Massachusetts, United States of America), MCSP (MAB 2029, MilliporeSigma, Missouri, United States of America), Ki67 (ab156956, Abcam, Cambridge, UK) and MelanA (ab731, Abcam, Cambridge, UK) were used as primary antibodies. Secondary antibodies were donkey anti-guinea pig Alexafluor 647, donkey anti-rabbit Alexafluor 488, anti-rat Alexafluor 594 and anti-mouse Alexafluor 594 (Invitrogen, Carlsbad, United States of America). Nuclear staining was performed with DAPI (Sigma-Aldrich, Taufkirchen, Germany). Samples were observed with a fluorescence microscope (BX51and DP80, Olympus, Tokyo, Japan) using cellSens software (Olympus, Tokyo, Japan) and Zeiss LSM 880 (Zeiss, Oberkochen, Germany).

### Immunoblotting

Keratinocytes were cultured to confluence with or without OR10A6 siRNA in 24-well culture plates and then homogenized in RIPA buffer (Nacalai tesque Inc., Kyoto, Japan) containing protease inhibitor cocktail (Nacalai tesque Inc., Kyoto, Japan). Protein concentrations were measured with a protein assay kit (BIO-RAD, CA, United States of America). Samples were subjected to sodium dodecyl sulfate polyacrylamide gel electrophoresis (Invitrogen, Carlsbad, United States of America) and transferred to PVDF membranes (Invitrogen, Carlsbad, United States of America). For the membrane staining, antibodies to OR10A6 (DF5047, Affinity Biosciences, Cincinnati, United States of America), Phospho-Erk1/2 (9101, cell signaling, Massachusetts, United States of America), Erk1/2 (9102, cell signaling, Massachusetts, United States of America), Phospho-JNK1/2/3 (9251, cell signaling, Massachusetts, United States of America), JNK1/2/3 (ab179461, Abcam, Cambridge, UK), Phospho-PLC-γ1 (8713, cell signaling, Massachusetts, United States of America), PLC-γ1 (5690, cell signaling, Massachusetts, United States of America) and α-tubulin (017–25031, FUJIFILM Wako Pure Chemical Corporation, Osaka, Japan) were used as primary antibodies and rabbit IgG HRP-linked F(ab’)2 antibody (NA9340V, GE Healthcare, UK) and Goat anti-mouse IgG (H + L) Secondary Antibody HRP (62–6520, Invitrogen, Carlsbad, United States of America) were used as secondary antibodies. The protein was detected using Chemi-Lumi One Super (Nacalai tesque Inc., Kyoto, Japan).

## Statistics

The statistical significance of differences among multiple groups was determined by ANOVA with Scheffé's method. *p* < 0.05 was considered significant. Student’s t*-*test was used to determine the significance of differences between two groups.

## Results

### Distinct expression patterns of OR genes associated with different human tissues

**FIGURE 1:**
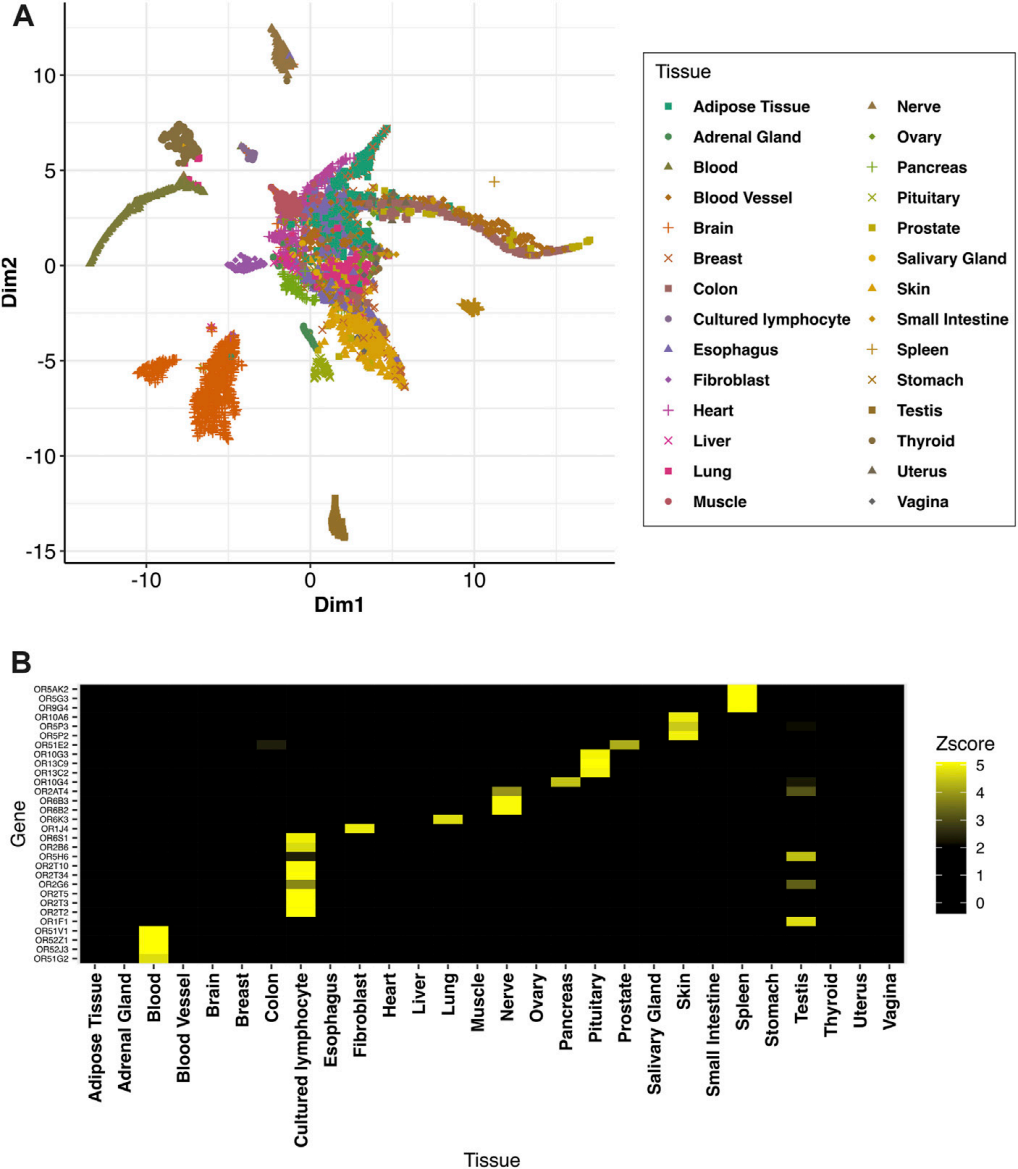
UMAP visualization and distinct OR genes highly expressed associate with different human tissues. **(A)** UMAP visualization for all samples in GTEx using 404 OR genes. The color and shape of each dot refers to the tissue recorded in the GTEx dataset. **(B)** OR genes characteristically expressed in different tissues. The color gradient represents the z-score for median expression of each gene per tissue. Since the testis shows a large number of characteristically expressed OR genes compared with other tissue, testis information was partially removed for clarity. Tissue identication is defined in the method section.

First, we set out to find OR genes characteristically expressed in different tissues by means of meta-transcriptome analysis of the RNA-sequencing data in the GTEx data set. As genes relevant to olfactory signaling, we picked up 404 OR genes listed in the KEGG pathway (hsa04740) or Reactome pathway (R-HSA-381753) for further analysis. To check whether the ORs show tissue-dependent gene expression patterns, we utilized UMAP, which is an un-supervised clustering method (Figure 1A). Most samples were clustered by tissue, but not by age, sex, or cause of death (Supplementary Figure S1A-D), supporting the idea that OR expression patterns are tissue-dependent. Next, the combination of the Wilcoxon rank sum test, fold expression per tissue and LDA was used to define the distinct expression patterns. OR genes highly expressed in at least one tissue according to our criteria are shown in Figure 1B. Since testis shows the highest number of highly expressed OR genes compared with other tissues (Supplementary Figure S1E), some of them were removed from Figure 1B for clarity. All data are shown in Supplementary Figure S2. Finally, we identified distinct sets of ORs that were highly expressed in 12 tissues, involving 97 ORs in total (Figure 1B, Supplementary Figure S2).

### Possible OR functions in skin

**FIGURE 2:**
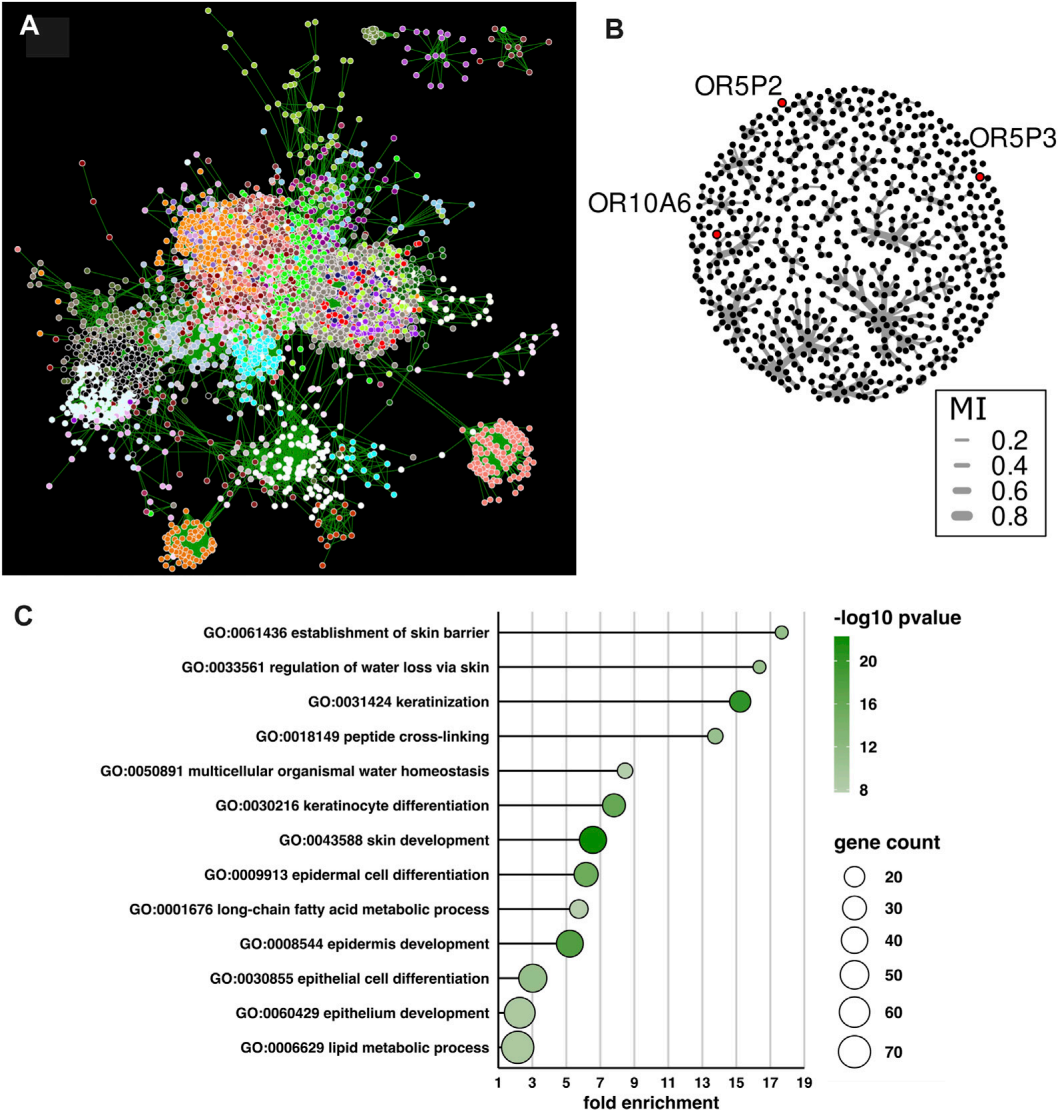
WGCNA and c3net analysis of genes expressed in skin tissue. **(A)** The gene co-expression network using the WGCNA algorithm for genes expressed in skin tissue was visualized using Cytoscape. Each node and edge correspond to gene and TOM similarity, respectively. Only edges for TOM similarity higher than 0.025 are visualized. Nodes without edges higher than TOM similarity 0.025 were removed. The color of each node corresponds to WGCNA modules. **(B)** Sub-network structure of the skin-associated OR-containing module, i.e., the black module. All genes in the black module were visualized using the c3net algorithm, which utilizes maximum mutual information for each gene. Edge width corresponds to mutual information between genes. **(C)** Gene ontology term enrichment analysis of the black module in WGCNA clustering.

Among the OR genes, *OR5P2*, *OR5P3* and *OR10A6* are characteristically expressed in skin tissue. To examine the functions of these genes, we employed WGCNA to identify gene network co-expression modules (van Dam et al., 2018). We applied WGCNA to all expressed genes in skin samples, except fibroblasts. In Figure 2A, we show the network based on a TOM similarity of more than 0.025 (Figure 2A). In each node, the color represents a gene module detected by WGCNA. All three ORs belonged to the black module. To further segment the genes in this module, we employed the c3net method, which utilizes maximum mutual information. Even though the three OR genes belong the same module, they belong to different sub-networks in the c3net network (Figure 2B). To infer what biological process these ORs are involved in, we applied gene ontology term enrichment analysis to the black module genes (Figure 2C). The results indicated that genes in this module are involved in epidermal differentiation and water-impermeable barrier homeostasis. In addition, genes in the black module were more highly expressed in skin than in other GTEx tissues (Supplementary Figure S3). Keratinocytes seem a promising candidate for the cell type expressing these ORs.

### OR10A6 expression in human keratinocytes and skin

**FIGURE 3:**
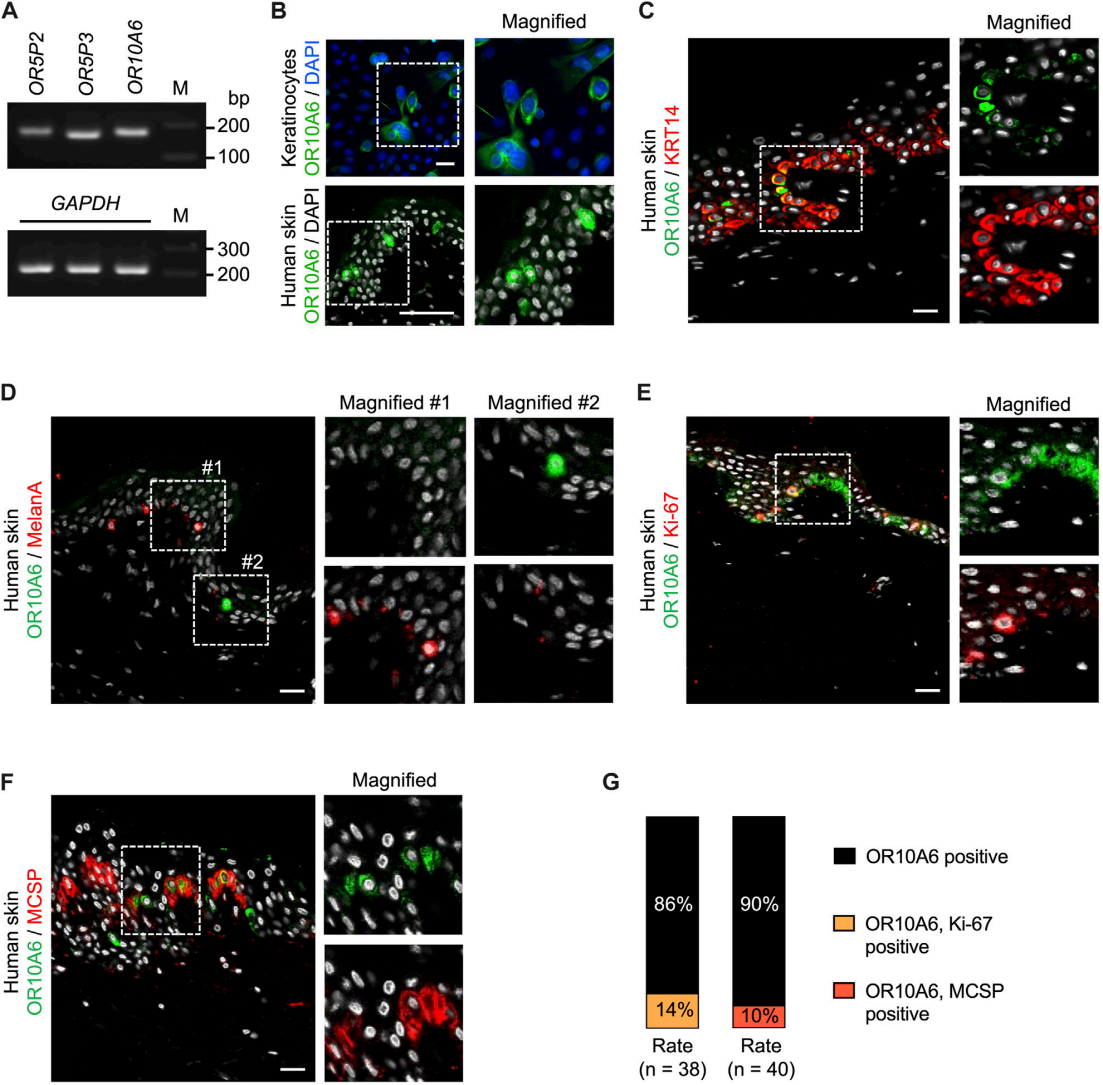
Expression of ORs in human keratinocytes and skin. **(A)** RT-PCR analysis of *OR5P2*, *OR5P3*, and *OR10A6*. *GAPDH* was used for RNA quality control. M: Marker. **(B)** Immunofluorescence staining of OR10A6 (green) in human keratinocytes and skin. Cell nuclei appear in blue or white. Magnified sections are indicated by white dashed squares. Bar = 50 μm. **(C–F)** Immunofluorescence staining of OR10A6 (green) and KRT14, Melan A, Ki-67 or MCSP (red) in human skin. Cell nuclei appear in white. Magnified sections are shown in white dashed squares. Bar = 20 μm. **(G)** Ratio of OR10A6-positive cells, OR10A6 + Ki-67-positive cells and OR10A6 + MCSP-positive cells counted in skin sections.

Next, we examined the expression of *OR5P2*, *OR5P3*, and *OR10A6* in keratinocytes and skin sections. RT-PCR analysis confirmed the expression of all three ORs (Figure 3A). Since *OR10A6* showed the largest gene sub-network in the c3net network (Figure 2B), we focused on OR10A6 for further study. OR10A6 was immunochemically detected sparsely in human keratinocytes (Figure 3B), and a similar expression pattern of OR10A6 was observed in skin sections (Figure 3B). The percentage of OR-positive cells in cultured keratinocytes was 31 ± 2%. In skin sections, OR10A6 was expressed in basal keratinocytes and the percentage of OR-positive cells to total basal keratinocytes was 9 ± 5%. Co-staining of OR10A6, KRT14, MelanA, MCSP, and Ki-67 showed that OR-positive cells overlapped with KRT14-positive cells but did not overlap with MelanA-positive cells (Figures 3C,D). They also partially, but not completely, overlapped with MCSP- or Ki-67-positive cells (Figures 3E–G).

### 3PPP-induced calcium response and CE production in keratinocytes

**FIGURE 4:**
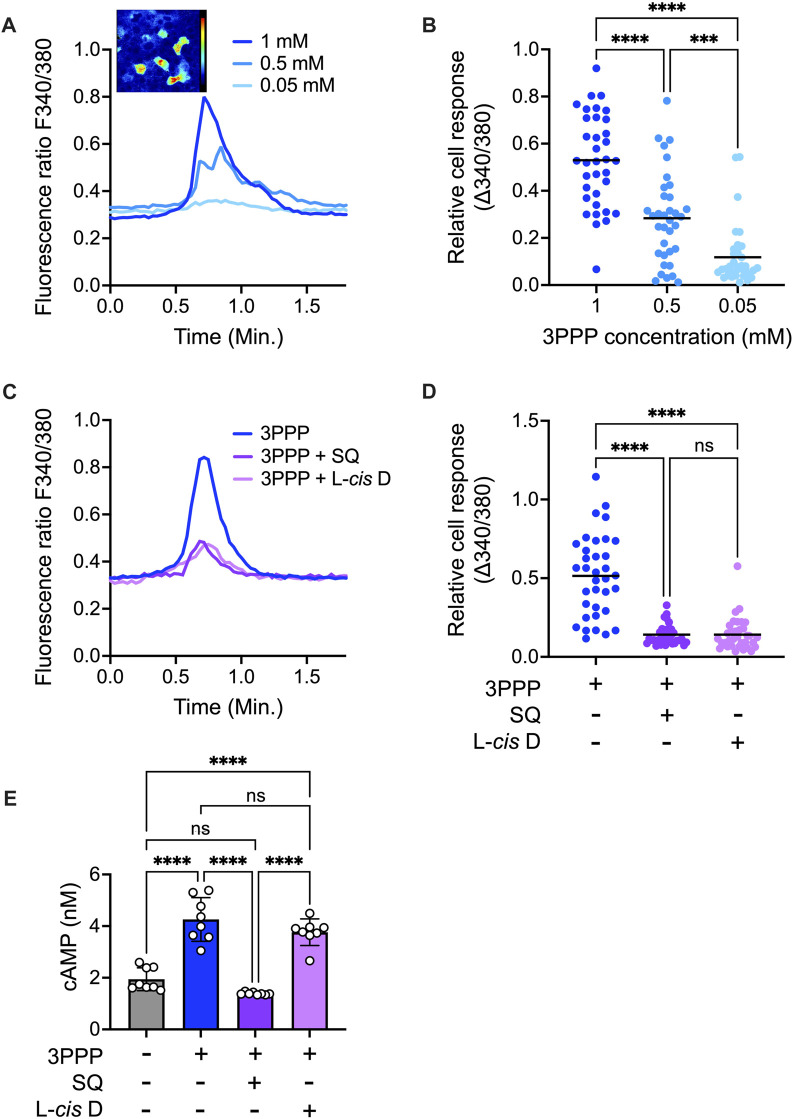
Calcium response and cAMP production induced by 3PPP in keratinocytes. **(A–C)** Representative profiles of intracellular calcium after 3PPP (1 mM for **(C)**) application with or without SQ-22536 (100 μM) and L-*cis*-diltiazem (100 μM) to the cells. Representative image of cells at 0.75 min in condition of 3PPP (1 mM) application is indicated in **(A)**. **(B–D)** Quantitation of fluorescence ratio change after application of 3PPP (1 mM for **(D)**) with or without SQ-22536 (100 μM) and L-*cis*-diltiazem (100 μM) to the cells. Anova F value = 49.38 and *p* < 0.0001 for **(B)**, Anova F value = 59.58 and *p* < 0.0001 for **(D)** (*n* = 35 cells). **(E)** cAMP measurement after application of 3PPP (1 mM) with or without SQ-22536 (100 μM) and L-*cis*-diltiazem (100 μM) to the cells. Anova F value = 52.38 and *p* < 0.0001 (*n* = 8). Similar results were obtained in three independent experiments. Bars and lines represent mean ± SD. ***: *p* < 0.0005, ****: *p* < 0.0001.

**FIGURE 5:**
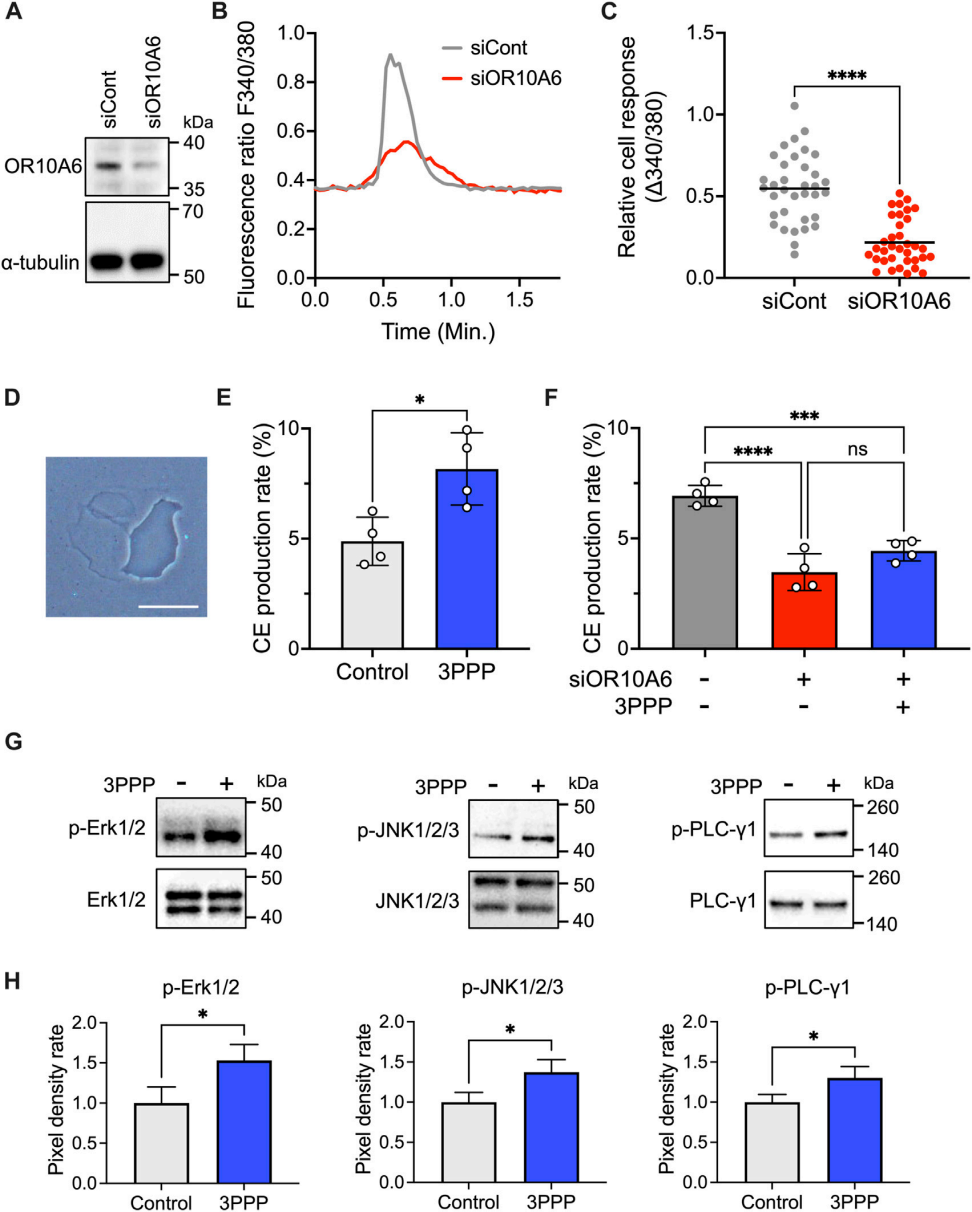
Calcium response, CE production and phosphokinase analysis induced by 3PPP in keratinocytes treated with OR10A6 siRNA. **(A)** Western blot analysis for the detection of OR10A6 in cells treated with scramble RNA or OR10A6 siRNA. **(B)** Representative profiles of intracellular calcium after 3PPP (1 mM) application to cells treated with scramble RNA or OR10A6 siRNA. **(C)** Quantitation of fluorescence ratio change after application of 3PPP (1 mM) to cells treated with scramble RNA or OR10A6 siRNA (n = 35 cells). **(D)** Representative image of produced CE. Bar = 50 μm. **(E)** CE production rate of keratinocytes treated with 3PPP (1 mM) (*n* = 4). **(F)** CE production rate of keratinocytes treated with scramble RNA or OR10A6 siRNA and 3PPP (1 mM). Anova F value = 33.63 and *p* < 0.0001 (*n* = 4). Similar results were obtained in three independent experiments. **(G)** Western blot analysis for the detection of phosphorylation of JNK1/2/3 and PLC-γ1 in cells treated with 3PPP (1 mM). **(H)** Quantitation of western blot signal (*n* = 3). Bars and lines represent mean ± SD. *: *p* < 0.05, ***: *p* < 0.001, ****: *p* < 0.0001.

Application of 3PPP, an agonist of OR10A6 (Mainland et al., 2015), to keratinocytes elevated the intracellular calcium concentration (Figure 4A). We defined responding cells as that with Δ340/380 > 0.015, and 33 ± 5% of total cells responded to 3PPP in a dose-dependent manner (Figures 4A,B). The concentration range was set by referencing previous reports (Xu et al., 2006; Klein et al., 2013; Lee et al., 2015). The 3PPP-induced calcium elevation was significantly reduced by co-application of the adenylyl cyclase inhibitor SQ-22536 or the cyclic nucleotide-gated (CNG) channel blocker L-*cis*-diltiazem (Figures 4C,D). Elevation of intracellular cAMP level was also observed following application of 3PPP, and this elevation was inhibited by co-application of SQ-22536 (Figure 4E). To confirm the role of OR10A6 in the 3PPP-induced calcium elevation, a knock-down study using siRNA targeting OR10A6 was performed. Reduction of OR10A6 expression was confirmed by western-blot analysis (Figure 5A). The 3PPP-induced calcium elevation was significantly reduced in OR10A6 siRNA-treated keratinocytes (Figures 5B,C).

Since OR10A6 was expressed in the basal layer of human skin (Figure 3B) and the black module genes, including OR10A6, are involved in epidermal differentiation processes (Figure 2C), we examined the relationship between OR10A6 and differentiation. In cell proliferation assay, we could not detect any significant difference between 3PPP-treated and control cells (data not shown). However, application of 3PPP significantly increased CE production in keratinocytes (Figures 5D,E). In addition, the CE production was significantly decreased in OR10A6 siRNA-treated keratinocytes, and application of 3PPP did not stimulate CE production in these cells (Figure 5F).

We also examined signaling factors potentially involved in the 3PPP-induced CE production by means of human phosphokinase array analysis. 37 kinase phosphorylation sites and 2 related proteins were measured (Supplementary Figure 4A) and JNK1/2/3 and PLC-γ1 phosphorylation sites were significantly changed in response to 3PPP treatment (Supplementary Figure 4B). Consistent with the array results, significant phosphorylation changes of JNK1/2/3 and PLC-γ1 response to 3PPP treatment were observed in western blot experiments (Figures 5G,H). Erk1/2 phosphorylation was also significantly changed with 3PPP treatment (Figures 5G,H).

## Discussion

In accordance with previous studies, we found different expression patterns of OR genes among different human tissues (Feldmesser et al., 2006; Flegel et al., 2013), and UMAP clustering showed that tissue was a better explanatory factor for the expression differences than sex, age or cause of death. The detection of a larger number of testis-associated ORs, compared with other tissues, is also consistent with previous studies (Feldmesser et al., 2006; Fukuda and Touhara, 2006; Flegel et al., 2013). Furthermore, we employed WGCNA and c3net analysis to visualize and analyze co-expression of the set of OR genes associated with skin.

*OR5P2*, *OR5P3*, and *OR10A6* were detected in RT-PCR analysis of human skin, and OR10A6 was observed at the protein level. We observed sparse expression of OR10A6 in human skin, and the OR-expressing cells were keratinocytes, but not melanocytes, because they were KRT14-positive and not MelanA-positive. Similar sparse expression of OR has been reported in human skin and human retina (Jovancevic et al., 2017b; Tsai et al., 2017). In contrast, broad expression of OR2AT4 was reported in human skin (Busse et al., 2014). Different ORs recognize different ranges of ligands (Reisert and Restrepo, 2009), and this may be related to the different expression patterns of ORs.

Sparse 3PPP-induced calcium responses in keratinocytes were observed and those results did not discrepancy with the immunochemical results. The pathway of olfactory signal transduction has been elucidated, and ORs transmit information to a G protein, G_olf_, which activates adenylyl cyclase. cAMP produced by adenylyl cyclase opens the CNG channel, resulting in an influx of Ca^2+^ and Na^+^ (Kaupp, 2010). These transduction proteins are also expressed in keratinocytes (Busse et al., 2014). We found that the adenylyl cyclase inhibitor SQ-22536 and the CNG channel blocker L-*cis*-diltiazem significantly inhibited the 3PPP-induced calcium response in keratinocytes. Significant cAMP level elevation was also induced by 3PPP, and the elevation was inhibited by SQ-22536. OR10A6 mediation of 3PPP-induced calcium response in keratinocytes was also confirmed by OR10A6 knock-down experiments. Similar results have been reported in other non-olfactory tissues (Spehr et al., 2004; Griffin et al., 2009; Pluznick et al., 2009; Busse et al., 2014; Kalbe et al., 2016; Tsai et al., 2017; Weidinger et al., 2021).

As regards downstream signaling, human phosphokinase array analysis and western blot experiments revealed that 3PPP significantly upregulated phosphorylation of Erk1/2 and JNK1/2/3. These phosphorylation cascades are known to promote keratinocyte differentiation (Takahashi et al., 1998; Sayama et al., 2001; Seo et al., 2004). Significant upregulation of PLC-γ1 was also observed in keratinocytes treated with 3PPP. Previous studies indicated that those proteins enhance the Ca^2+^ sensitivity of keratinocytes and promote keratinocyte differentiation (Xie and Bikle, 1999).

In line with those results, the black module genes are involved in epidermal differentiation processes, and application of 3PPP significantly stimulated CE production of keratinocytes. The CE stimulation was diminished by OR10A6 siRNA treatment. Since endogenous substances recognized by ORs include short- and medium-chain fatty acids and androstenone (Keller et al., 2007; Hartmann et al., 2013; Rooks and Garrett, 2016; Jovancevic et al., 2017a), OR10A6 may recognize endogenous substances that regulate general differentiation processes. Although OR10A6-expressing cells were localized in the basal layer, the application of 3PPP did not change the proliferation of keratinocytes, in accordance with the results of co-staining of ORs, MCSP, and Ki-67, which indicated that OR-expressing cells are not identical with proliferating cells. Our results suggest that sparse expression of OR10A6 cells in the basal layer is important for epidermal differentiation. However, future research will be required to understand the elaborate cell-cell communication that underpins homeostasis in the skin. Although availability of single-cell transcriptome data has recently been increasing, the read depth of mRNA is generally insufficient for OR transcription analysis. Improvement of single-cell transcriptome technology may lead to a better understanding of OR functions in the skin, as well as the heterogeneity of keratinocytes.

Overall, integration of meta-transcriptome analysis and functional analysis uncovered distinct sets of ORs that were highly expressed in 12 tissues, involving 97 ORs in total. Those results provide helpful information for future studies of distinctly expressed ORs associated with various tissues. We found that *OR5P2*, *OR5P3* and *OR10A6* were characteristically expressed in skin. Furthermore, we confirmed that OR10A6 activation by the agonist 3PPP stimulated CE production. Our results suggest that OR10A6 might be a therapeutic target to modulate keratinization.

## Data Availability

Publicly available datasets were analyzed in this study. This data can be found here: https://gtexportal.org/home/datasets.
