# Supplementary material for: Distinct sets of olfactory receptors highly expressed in different human tissues evaluated by meta-transcriptome analysis: Association of OR10A6 in skin with keratinization
Source: Front Cell Dev Biol. 2023 Jan 26;11:1102585. doi: 10.3389/fcell.2023.1102585 (PMC9909485; doi:10.3389/fcell.2023.1102585)
Supplement: Supplementary file 1 [file DataSheet1.docx]

Supplementary Material


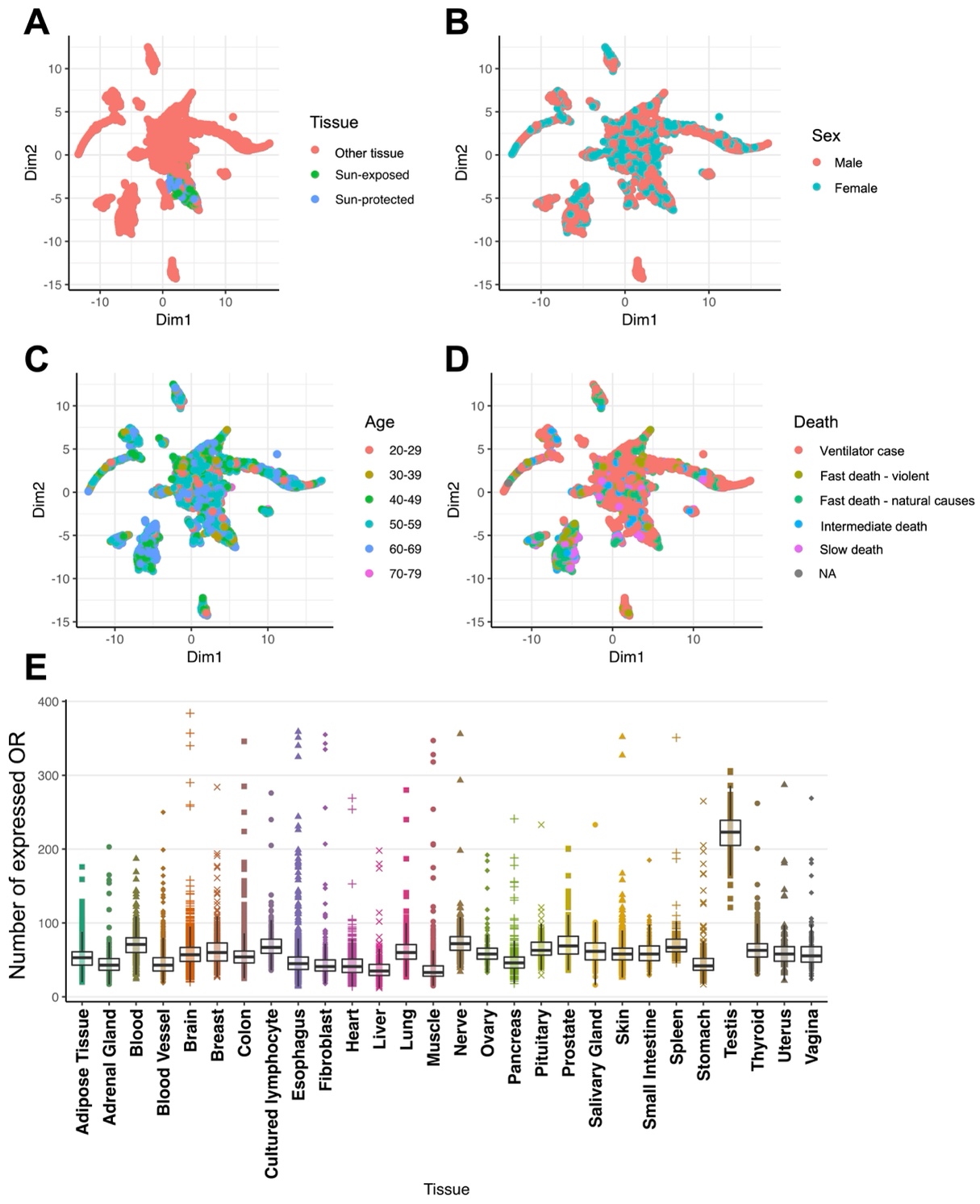


**Supplementary Figure 1.** UMAP visualization and number of ORs expressed in each tissue.

**(A)** The colors of the dots refer to tissues. Skin tissue is shown in green (sun-exposed) or blue (sun-protected). **(B-D)** The colors refer to sex, age or cause of death. **(E)** Number of OR genes for which expression level >0.


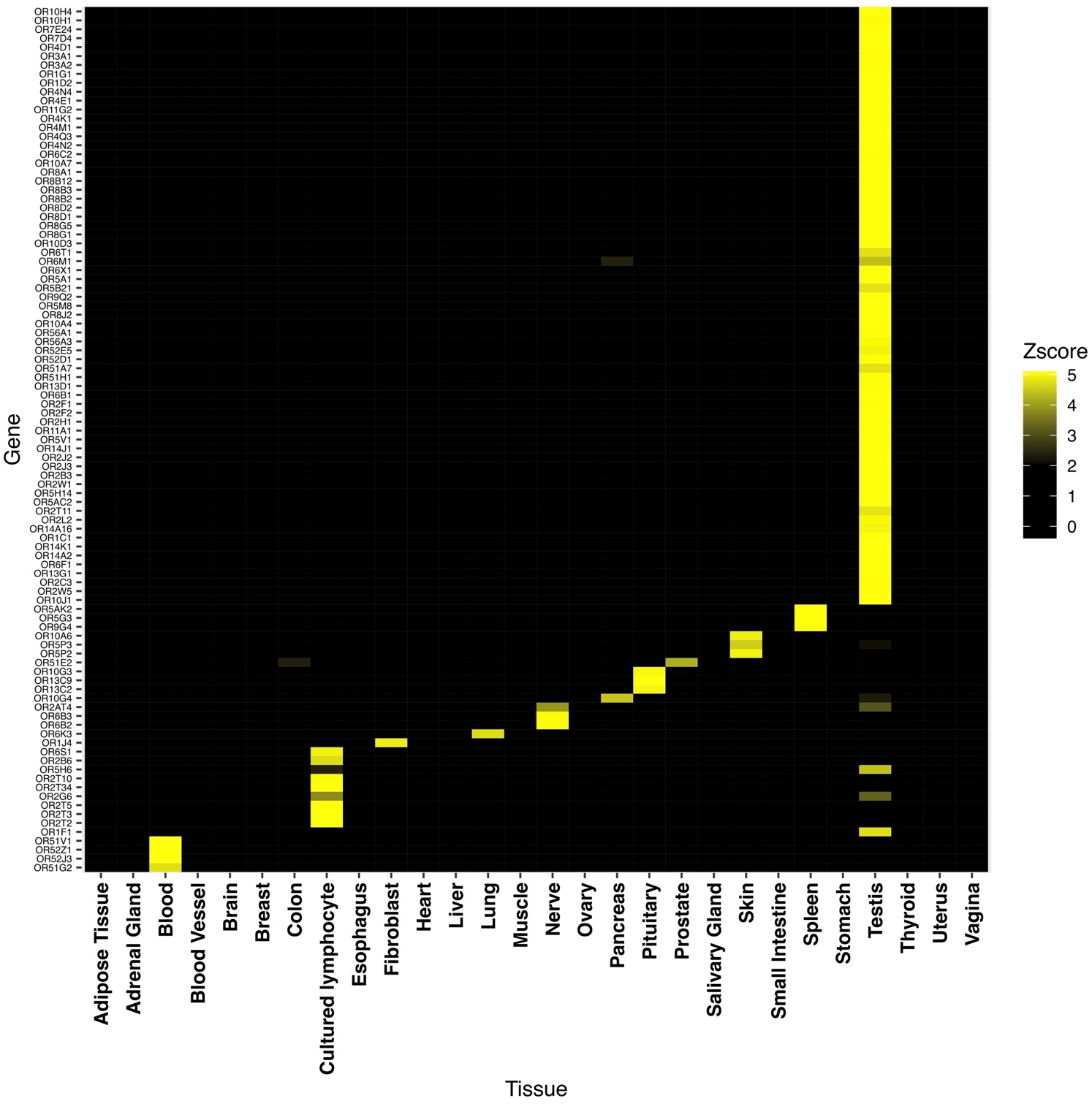


**Supplementary Figure 2.** Distinct OR genes highly expressed associated with various tissues.

The color gradient indicates the z-score for median expression of each gene per tissue.


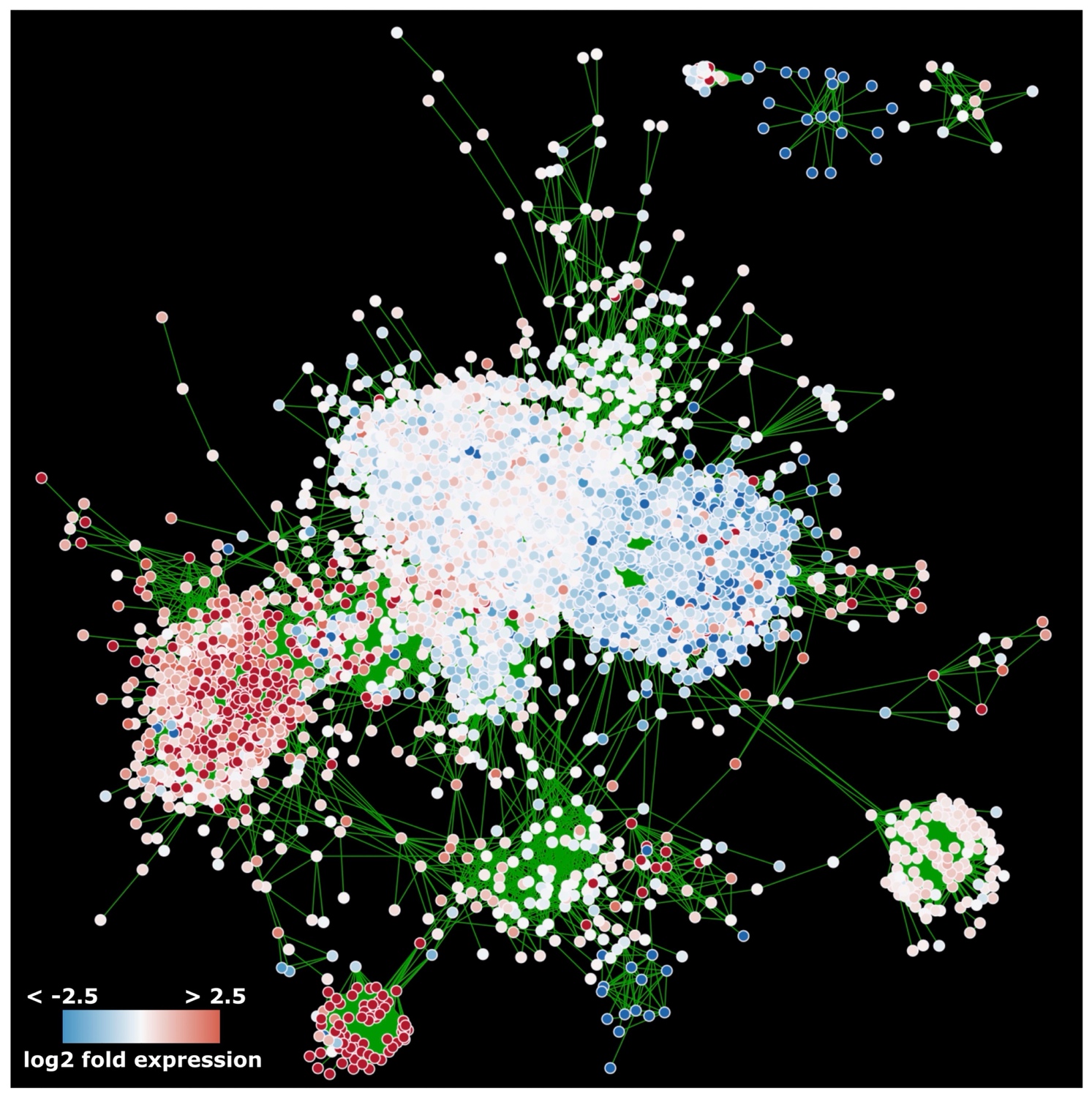


**Supplementary Figure 3.** Gene co-expression network obtained using the WGCNA algorithm.

Colors indicate log2-fold expression for each gene in the skin tissue compared with other tissues.


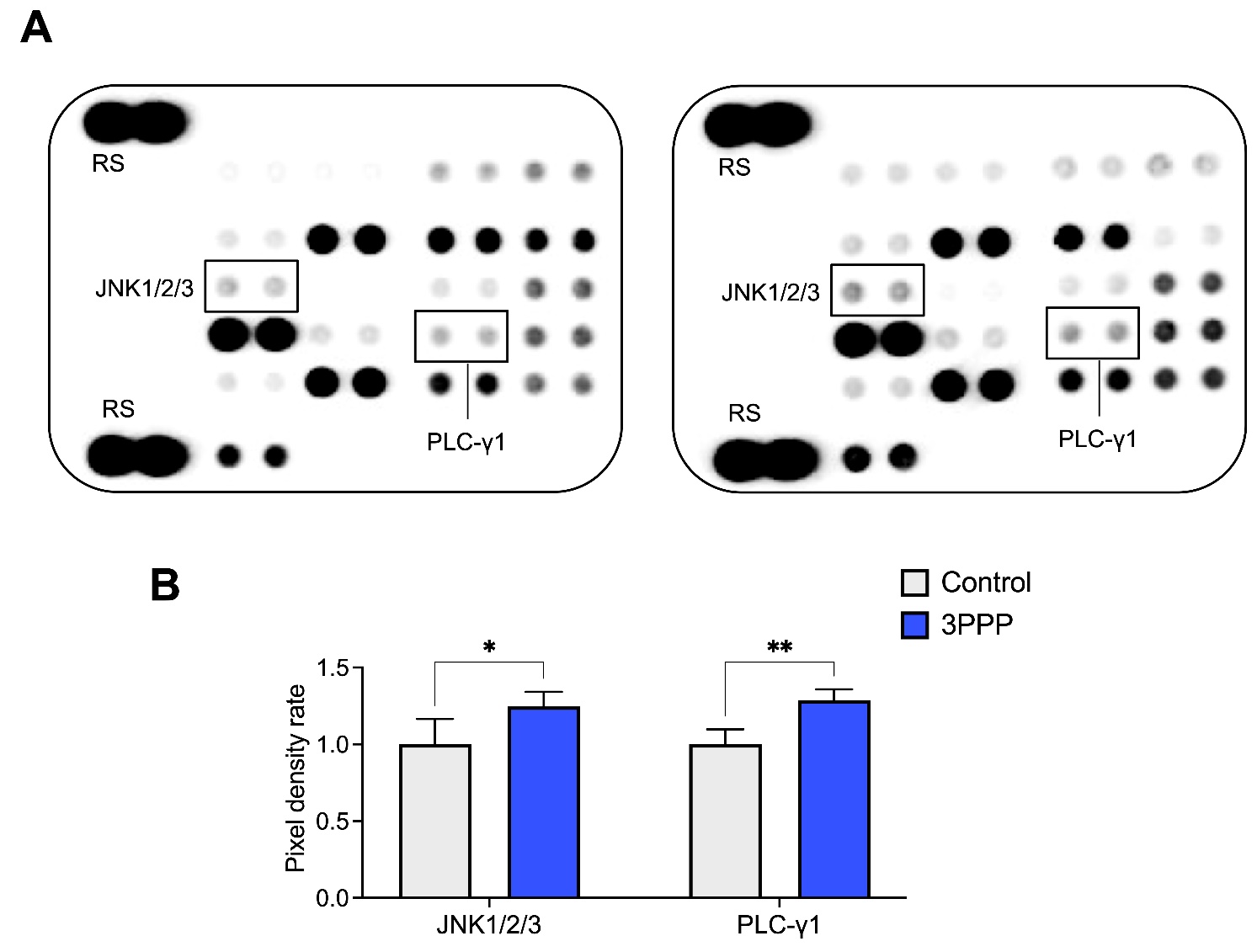


**Supplementary Figure 4.** Phosphokinase array analysis of keratinocytes treated with 3PPP.

**(A)** Representative image of array results of cell lysates treated with 3PPP (1 mM). RS: positive reference spot. **(B)** Quantitation of JNK 1/2/3 and PLC-γ1array spot signal (n = 4). Bars and lines represent mean ± SD. * : *P* < 0.05, ** : *P* < 0.01.
